# Supplementary material for: Identification of photoperiod-induced specific miRNAs in the adrenal glands of Sunite sheep (Ovis aries)
Source: Front Vet Sci. 2022 Jul 22;9:888207. doi: 10.3389/fvets.2022.888207 (PMC9354845; doi:10.3389/fvets.2022.888207)
Supplement: Supplementary file 1 [file Data_Sheet_1.ZIP › Supplementary file/miRDeep2 file.zip!/miRDeep2 file/Novel_Structure/SPLP42A1/Novel_697.pdf]

Provisional ID	:	NC_019483.2_31295
Score total	:	18.1
Score for star read(s)	:	3.9
Score for read counts	:	10.3
Score for mfe	:	2.3
Score for randfold	:	1.6
Score for cons. seed	:	
Total read count	:	32
Mature read count	:	31
Loop read count	:	0
Star read count	:	1

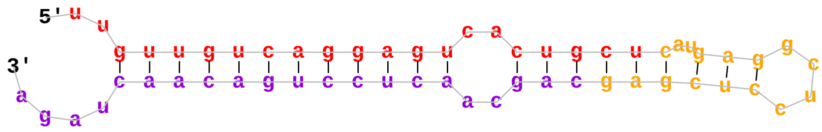

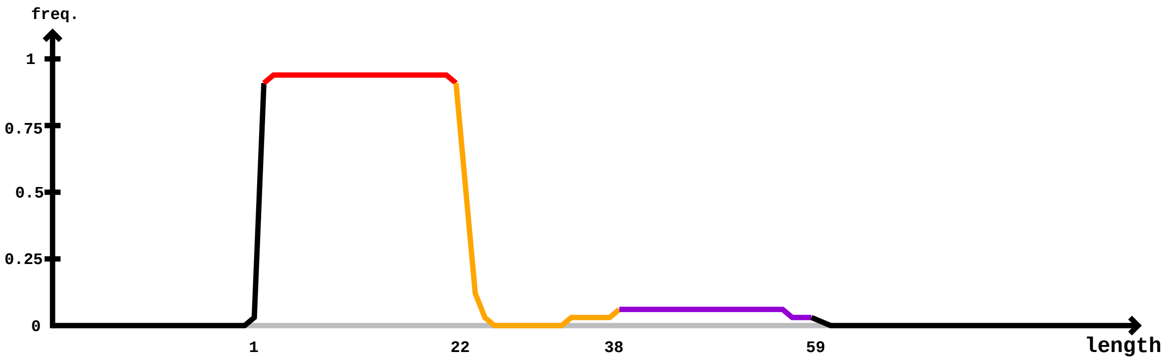


```
                     Mature                                  Star
5'- gacgauguaggcucacccacuuguugucaggagucacugcucaugaggcuccucgagcagcaacuccugacaacuagaagggagggcagagcaucagcgcagccccugugg   -3'   obs
    gacgauguaggcucacccacuuguugucaggagucacugcucaugaggcuccucgagcagcaacuccugacaacuagaagggagggcagagcaucagcgcagccccugugg         exp
    ...(((((..((((.(((.((.((((((((((((..((((((..(((....)))))))))..))))))))))))).))..))).))))...)))))..(((((...))))).    reads    mm    sample
    ...................cuuguugucaggagucacugcu......................................................................    1        0     seq
    ....................uuguugucaggagucacugc.......................................................................    1        0     seq
    ....................uuguugucaggagucacugcu......................................................................    14       0     seq
    ....................uuguugucaggagucacugcuc.....................................................................    10       0     seq
    ....................uuguugucaggagucacugcuca....................................................................    3        0     seq
    ....................uuguugucaggagucacugcucau...................................................................    1        0     seq
    .....................uguugucaggagucacugcuc.....................................................................    1        0     seq
    ....................................................ucgagcagcaacuccugacaacu....................................    1        0     seq
    .........................................................cagcaacuccugacaacuaga.................................    1        0     seq
```
